# Supplementary material for: Modeling glioblastoma heterogeneity as a dynamic network of cell states
Source: Mol Syst Biol. 2021 Sep 16;17(9):e10105. doi: 10.15252/msb.202010105 (PMC8444284; doi:10.15252/msb.202010105)
Supplement: Supplementary file 6 — Source Data for Figure 5 [file MSB-17-e10105-s004.zip › Figure5A_sourcedata/GSEA_3071/hallmarks_stateII.GseaPreranked.1623742536692/HALLMARK_OXIDATIVE_PHOSPHORYLATION.html]

Details for gene set HALLMARK\_OXIDATIVE\_PHOSPHORYLATION[GSEA]

|  || Dataset | state23071 |
| Phenotype | NoPhenotypeAvailable |
| Upregulated in class | na\_neg |
| GeneSet | HALLMARK\_OXIDATIVE\_PHOSPHORYLATION |
| Enrichment Score (ES) | -0.22770086 |
| Normalized Enrichment Score (NES) | -1.1878482 |
| Nominal p-value | 0.22091062 |
| FDR q-value | 0.5457283 |
| FWER p-Value | 0.999 |
Table: GSEA Results Summary

  

Fig 1: Enrichment plot: HALLMARK\_OXIDATIVE\_PHOSPHORYLATION      
 Profile of the Running ES Score & Positions of GeneSet Members on the Rank Ordered List

  

| PROBE | GENE SYMBOL | GENE\_TITLE | RANK IN GENE LIST | RANK METRIC SCORE | RUNNING ES | CORE ENRICHMENT || 1 | IDH2 |  |  | 78 | 1.117 | 0.0191 | No |
| 2 | SLC25A5 |  |  | 680 | 0.537 | -0.1115 | No |
| 3 | TIMM9 |  |  | 689 | 0.535 | -0.0950 | No |
| 4 | CYB5A |  |  | 737 | 0.520 | -0.0888 | No |
| 5 | FH |  |  | 849 | 0.485 | -0.0996 | No |
| 6 | CYCS |  |  | 1113 | 0.415 | -0.1506 | No |
| 7 | MRPS15 |  |  | 1226 | 0.392 | -0.1649 | No |
| 8 | LDHA |  |  | 1374 | 0.361 | -0.1889 | No |
| 9 | GLUD1 |  |  | 1397 | 0.358 | -0.1820 | No |
| 10 | IMMT |  |  | 1436 | 0.350 | -0.1794 | No |
| 11 | MRPL15 |  |  | 1477 | 0.343 | -0.1775 | No |
| 12 | GRPEL1 |  |  | 1531 | 0.335 | -0.1791 | No |
| 13 | NQO2 |  |  | 1563 | 0.330 | -0.1754 | No |
| 14 | TIMM10 |  |  | 1567 | 0.329 | -0.1648 | No |
| 15 | FXN |  |  | 1663 | 0.315 | -0.1775 | No |
| 16 | ATP5MC1 |  |  | 1769 | 0.302 | -0.1931 | No |
| 17 | LRPPRC |  |  | 1836 | 0.294 | -0.1994 | No |
| 18 | AFG3L2 |  |  | 1840 | 0.293 | -0.1900 | No |
| 19 | MRPL11 |  |  | 1854 | 0.292 | -0.1832 | No |
| 20 | OGDH |  |  | 1855 | 0.291 | -0.1732 | No |
| 21 | HSPA9 |  |  | 1884 | 0.287 | -0.1702 | No |
| 22 | CYC1 |  |  | 1938 | 0.281 | -0.1737 | No |
| 23 | CPT1A |  |  | 1959 | 0.278 | -0.1691 | No |
| 24 | ATP6V1D |  |  | 1963 | 0.277 | -0.1602 | No |
| 25 | OXA1L |  |  | 1969 | 0.277 | -0.1519 | No |
| 26 | SURF1 |  |  | 2223 | -0.250 | -0.2061 | No |
| 27 | PHYH |  |  | 2249 | -0.254 | -0.2036 | No |
| 28 | ATP5ME |  |  | 2320 | -0.261 | -0.2119 | No |
| 29 | POR |  |  | 2321 | -0.261 | -0.2029 | No |
| 30 | COX7B |  |  | 2400 | -0.271 | -0.2129 | No |
| 31 | ATP1B1 |  |  | 2433 | -0.275 | -0.2114 | No |
| 32 | HADHB |  |  | 2489 | -0.283 | -0.2153 | No |
| 33 | ATP6V1E1 |  |  | 2540 | -0.292 | -0.2176 | Yes |
| 34 | ATP6V0E1 |  |  | 2545 | -0.293 | -0.2085 | Yes |
| 35 | ATP6V1C1 |  |  | 2606 | -0.302 | -0.2130 | Yes |
| 36 | NDUFB1 |  |  | 2611 | -0.303 | -0.2036 | Yes |
| 37 | COX6A1 |  |  | 2629 | -0.305 | -0.1973 | Yes |
| 38 | ATP5F1D |  |  | 2652 | -0.308 | -0.1921 | Yes |
| 39 | COX7C |  |  | 2660 | -0.310 | -0.1832 | Yes |
| 40 | NDUFAB1 |  |  | 2716 | -0.320 | -0.1858 | Yes |
| 41 | NDUFB7 |  |  | 2852 | -0.345 | -0.2074 | Yes |
| 42 | NDUFA1 |  |  | 2878 | -0.351 | -0.2015 | Yes |
| 43 | ECH1 |  |  | 2885 | -0.353 | -0.1909 | Yes |
| 44 | ATP6V1G1 |  |  | 2968 | -0.367 | -0.1986 | Yes |
| 45 | TIMM8B |  |  | 2980 | -0.369 | -0.1886 | Yes |
| 46 | ATP6AP1 |  |  | 3010 | -0.377 | -0.1828 | Yes |
| 47 | ALDH6A1 |  |  | 3027 | -0.379 | -0.1737 | Yes |
| 48 | COX5B |  |  | 3100 | -0.399 | -0.1778 | Yes |
| 49 | ISCU |  |  | 3101 | -0.399 | -0.1641 | Yes |
| 50 | MAOB |  |  | 3102 | -0.399 | -0.1503 | Yes |
| 51 | COX6B1 |  |  | 3120 | -0.404 | -0.1406 | Yes |
| 52 | NDUFB5 |  |  | 3158 | -0.415 | -0.1355 | Yes |
| 53 | BDH2 |  |  | 3162 | -0.418 | -0.1219 | Yes |
| 54 | ACAA1 |  |  | 3203 | -0.429 | -0.1170 | Yes |
| 55 | NDUFC1 |  |  | 3244 | -0.438 | -0.1118 | Yes |
| 56 | DECR1 |  |  | 3261 | -0.444 | -0.1005 | Yes |
| 57 | NDUFA2 |  |  | 3445 | -0.517 | -0.1281 | Yes |
| 58 | COX17 |  |  | 3461 | -0.524 | -0.1137 | Yes |
| 59 | UQCR11 |  |  | 3469 | -0.527 | -0.0973 | Yes |
| 60 | ATP6V0C |  |  | 3474 | -0.531 | -0.0800 | Yes |
| 61 | SLC25A4 |  |  | 3507 | -0.544 | -0.0692 | Yes |
| 62 | NDUFA7 |  |  | 3571 | -0.583 | -0.0647 | Yes |
| 63 | ATP5F1E |  |  | 3620 | -0.617 | -0.0554 | Yes |
| 64 | NDUFA3 |  |  | 3675 | -0.663 | -0.0460 | Yes |
| 65 | NDUFB4 |  |  | 3678 | -0.668 | -0.0234 | Yes |
| 66 | COX6C |  |  | 3792 | -0.782 | -0.0245 | Yes |
| 67 | IDH1 |  |  | 3842 | -0.832 | -0.0080 | Yes |
| 68 | GPX4 |  |  | 3915 | -0.986 | 0.0081 | Yes |
| 69 | PDP1 |  |  | 3954 | -1.075 | 0.0357 | Yes |
Table: GSEA details [plain text format]

  

Fig 2: HALLMARK\_OXIDATIVE\_PHOSPHORYLATION: Random ES distribution      
 Gene set null distribution of ES for **HALLMARK\_OXIDATIVE\_PHOSPHORYLATION**
